# Harmonizing healthcare and other resource measures for evaluating economic costs in substance use disorder research

**DOI:** 10.1186/s13011-021-00356-z

**Published:** 2021-04-08

**Authors:** Michelle A. Papp, Jared A. Leff, Sean M. Murphy, April Yang, Heidi M. Crane, Lisa R. Metsch, Carlos Del Rio, Daniel J. Feaster, Josiah D. Rich, Bruce R. Schackman, Kathryn E. McCollister

**Affiliations:** 1 Department of Population Health Sciences, Weill Cornell Medical College, 425 E 61st St, New York, NY 10065 USA; 2 Department of Public Health Sciences, University of Miami Miller School of Medicine, 1120 NW 14th St, Miami, FL 33136 USA; 3 Department of Infectious Diseases, University of Washington School of Medicine, Box 359931, Harborview Medical Center, 325 9th Ave, Seattle, WA 98104 USA; 4 Department of Sociomedical Sciences, Columbia University Mailman School of Public Health, 722 168th St, New York, NY 10032 USA; 5 Emory University School of Medicine, Grady Memorial Hospital, Emory Center for AIDS Research, 201 Dowman Drive, Atlanta, GA 30322 USA; 6 The Miriam Hospital, Brown University, 164 Summit Ave, Providence, RI 02906 USA

**Keywords:** Data harmonization, Economic evaluation, Substance use disorder, Healthcare resource utilization

## Abstract

**Background:**

Standardization and harmonization of healthcare resource utilization data can improve evaluations of the economic impact of treating people with substance use disorder (SUD), including reductions in use of expensive hospital and emergency department (ED) services, and can ensure consistency with current cost-effectiveness and cost-benefit analysis guidelines.

**Methods:**

We examined self-reported healthcare and other resource utilization data collected at baseline from three National Institute on Drug Abuse (NIDA)-funded Seek, Test, Treat, and Retain intervention studies of individuals living with/at risk for HIV with SUD. Costs were calculated by multiplying mean healthcare resource utilization measures by monetary conversion factors reflecting cost per unit of care. We normalized baseline recall timeframes to past 30 days and evaluated for missing data.

**Results:**

We identified measures that are feasible and appropriate for estimating healthcare sector costs including ED visits, inpatient hospital and residential facility stays, and outpatient encounters. We also identified two self-reported measures to inform societal costs (days experiencing SUD problems, participant spending on substances). Missingness was 8% or less for all study measures and was lower for single questions measuring utilization in a recall period.

**Conclusions:**

We recommend including measures representing units of service with specific recall periods (e.g., 6 months vs. lifetime), and collecting healthcare resource utilization data using single-question measures to reduce missingness.

**Supplementary Information:**

The online version contains supplementary material available at 10.1186/s13011-021-00356-z.

## Background

Economic evaluation is a critical tool to determine the value of substance use disorder (SUD) treatment. A 2017 report from the Council of Economic Advisors to the President highlighted the need for economic analyses to evaluate and improve the delivery of SUD treatment in the United States (U.S.) [1]. In the U.S., annual economic costs for opioid use disorder (OUD) alone are estimated at $787 billion, of which $89 billion represent healthcare resource utilization costs [2]. Reducing high-cost healthcare resource utilization is an important positive externality associated with effectively treating SUD that can generate significant savings to the healthcare sector. According to conservative estimates, $1 invested in SUD treatment yields a return of between $4 and $7 in reduced drug-related crime, criminal justice costs, and theft [3]. When savings to the healthcare sector are taken into account, total savings can exceed costs by a ratio of 12 to 1 [3].

An economic evaluation examines the cost of a prevention or treatment intervention, program, or policy in the context of potential downstream cost-offsets. These analyses leverage data across healthcare and non-healthcare domains [4]. National surveys such as the National Survey on Drug Use and Health and the National Longitudinal Survey on Adolescent Health include questions regarding number of visits to the emergency department (ED) and number of nights spent in the hospital [5, 6]. Many clinical effectiveness trials capture healthcare resource utilization through standardized instruments such as the Addiction Severity Index, Non-study Medical and Other Services, or Global Appraisal of Individual Needs [7–9]. While the healthcare service domain is common across many SUD comparative effectiveness studies, the specific measures, assessment timeframes, and responsiveness of participants vary substantially and can make conducting cross-study or integrative data analyses complicated or not feasible. Integrative data analysis is an important tool for analyzing pooled data from multiple studies to improve empirical capabilities and the robustness of findings [10]. Data harmonization applies common measures in order to improve the quality and comparability of data across independent studies, such that they can be synthesized to promote more rigorous and generalizable analyses of the impact of an intervention, program, or policy. It is especially important because new sources of data are available through electronic health records and insurance claims systems [11, 12].

Data harmonization in research pertaining to SUD, HIV, hepatitis C virus (HCV), and other related diseases and disorders is a high priority research area for the National Institute on Drug Abuse (NIDA) [13]. NIDA funded a large-scale prospective data collection and harmonization effort across 22 unique studies testing the Seek, Test, Treat, and Retain (STTR) model of HIV continuing care interventions for high-risk and hard-to-reach individuals with SUD [14, 15]. Self-reported healthcare resource utilization is a reliable proxy for medical claims and administrative data [16] and can be valued in dollars using monetary conversion factors (MCFs) found in published studies, government reports, and national data sets [17]. To inform data harmonization goals, we examined self-reported baseline data from three of the STTR studies to compare measures of healthcare resource utilization, evaluate the potential for combining these measures to estimate healthcare costs, and provide guidance for future studies on how to adopt healthcare resource utilization measures that are appropriate for economic evaluation.

## Methods

To gain access to individual-level, de-identified STTR study data, we submitted a concept proposal to the STTR Data Coordination Center at the University of Washington, which was reviewed and approved in 2016. Individuals interested in collaborating or working with these data should contact the STTR Data Coordination Center at sttr@uw.edu. For this study, we initially selected six STTR studies based on similarity across baseline questionnaires and relative completeness of de-identified baseline data. Three of these studies were later excluded because the baseline questionnaires collected healthcare resource utilization data without specific recall periods and could not be used to meaningfully calculate costs.

Collectively, 868 people living with HIV with or at high-risk for SUD are represented in three studies: (1) PACTo: Enhanced Access to HIV Care for Drug Users in San Juan, Puerto Rico, which implemented and evaluated a community-level, structured approach to 409 people living with HIV who use substances in five communities in San Juan from 2014 to 2017 [18]; (2) Project RETAIN: Providing Integrated Care for HIV-Infected Crack Cocaine Users, which evaluated the efficacy of an integrated HIV and primary care “retention clinic” in achieving virologic suppression compared to treatment as usual in 360 people living with HIV who used cocaine in Miami, FL and Atlanta, GA from 2013 to 2017 [19]; and (3) BRIGHT 2: Baltimore-Rhode Island Get HIV Tested, which evaluated the effectiveness of HIV linkage to care comparing intensive case management to treatment as usual in community corrections offices in 99 people living with HIV who were on probation or parole in Baltimore, MD from 2011 to 2015 [20, 21]. Healthcare resource utilization data were self-reported by study participants at baseline.

We identified healthcare resource utilization measures common to at least two of the selected studies with comparable recall periods, and categorized them into three domains: general medical care (e.g., hospital-based ED visits), SUD treatment (e.g., times treated for alcohol use disorder (AUD)), and medications (e.g., prescribed medication for AUD) (Table 1). We also included participant spending on substances, a measure shared by PACTo and RETAIN. Baseline healthcare resource utilization was reported across varying recall timeframes ranging from past 30 days to lifetime.

**Table 1:** Self-reported Measure Recall Periods in Three STTR Studies

Measure	PACTo	RETAIN	BRIGHT 2
*General medical care*			
Emergency department visits (hospital-based)	6 mo	6 mo	12 mo
Hospitalizations	6 mo	6 mo	12 mo
Hospital clinic / outpatient department visits	6 mo	6 mo	12 mo
Community clinic / outpatient department visits	6 mo	6 mo	12 mo
Physician visits	6 mo	6 mo	12 mo
Mental healthcare provider visits (psychological / emotional issues)	6 mo	6 mo	12 mo
Mental healthcare provider visits (medication management)	6 mo	6 mo	12 mo
Dental care visits	6 mo	6 mo	
Emergency dental care visits	6 mo	6 mo	
Provider visits for trauma counseling	6 mo	6 mo	
Nights in homeless or emergency shelter	6 mo	6 mo	
Case managers or case workers	6 mo	6 mo	12 mo
*SUD treatment*			
Times treated for alcohol use	lifetime	lifetime	lifetime
Times treated for substance use disorder	lifetime	lifetime	lifetime
Residential drug or alcohol treatment facility / detoxification hospital	6 mo	6 mo	12 mo
Alcohol / drug treatment provider visits	6 mo	6 mo	12 mo
Alcohol / drug treatment provider visits (medication management)	6 mo	6 mo	12 mo
*Medications*			
Prescribed medication for alcohol use disorder	30 days	30 days	lifetime
Prescribed medication for substance use disorder	30 days	30 days	lifetime
*Spending on substances*			
Money spent on drugs	30 days	30 days	
Money spent on alcohol	30 days	30 days	

Note: A blank cell confers the measure was not asked in that study

We reviewed common healthcare resource utilization measures to identify outcomes that are comparable across studies and could potentially be used for economic analyses. A prerequisite was that variables must represent units (e.g., number of hospital-based ED visits) over a specific recall period (e.g., last 30 days). Dichotomous measures such as “ever been treated for substance use disorder” or measures over lifetime cannot be meaningfully monetized for use in economic evaluations. We identified 10 measures that met this criteria and were representative of the healthcare sector perspective. These measures captured data on ED, inpatient hospital and residential facility, and outpatient encounters. Additional measures informed a broader, societal perspective by capturing reported number of days experiencing alcohol- or drug-related problems, and participant spending on alcohol or drugs in a given recall period. Some of these measures evaluated utilization during a specified time frame using response from a single question (e.g., number of hospital-based ED visits in a specific recall period) whereas other measures captured utilization using a combination of questions (e.g., number of hospitalizations in a specific recall period and number of days spent in the hospital per reported hospitalization) to calculate the total number of hospital days in the recall period.

We constructed descriptive statistics for each measure across all three studies (Table 2). To normalize different baseline assessment time-frames, we considered extrapolating data to the longest recall period (12 months). For instance, responses to measures reported “per 30 days” can be multiplied by 12 to represent a “per 12 month” measure. However, we instead created measures of the “average healthcare resource utilization per 30 days” by dividing 6-month and 12-month data by the represented number of months. While both adjustments rely on a limiting assumption that the rate of healthcare resource utilization remains constant over time, creating an average with real data points vs. adding data points through extrapolation was deemed a more conservative and preferred approach. Inpatient hospital days, residential facility days, and outpatient visits were calculated using a combination of the number of these events reported and the corresponding number of days per event. All studies collected data on the number of hospitalizations as well as the number of days per hospitalization (up to five most recent hospitalizations at baseline). In these instances, we created a measure of average event frequency (number of hospitalizations) per 30 days. If any individually-reported event-length (number of days spent in the hospital per hospitalization) exceeded 30 days, we adjusted to a 30-day maximum. We then multiplied the adjusted event frequency by the average event length in order to calculate a utilization measure that was translatable to dollars (e.g., number of days spent in the hospital per 30-day period).

**Table 2:** Mean Self-reported Healthcare Resource Utilization in Past 30 Days at Study Baseline

Measure	PACTo		RETAIN		BRIGHT 2	
	Mean	SD	Mean	SD	Mean	SD
**Healthcare sector perspective**						
**# of emergency department visits (hospital-based)**	**0.069**	**0.174**	**0.099**	**0.195**	**0.107**	**0.160**
* Inpatient*						
* Hospitalizations*						
# of hospitalizations	0.044	0.155	0.129	0.745	0.055	0.095
# of days per hospitalization	8.837	8.740	8.110	7.968	5.712	6.119
**# of hospitalization days**	**0.391**	**1.669**	**0.742**	**3.437**	**0.313**	**0.671**
*Residential drug or alcohol treatment facility / detox hospital*						
# of residential drug or alcohol treatment facility / detox hospital stays	0.077	0.340	0.039	0.300	0.013	0.033
# of days per residential drug or alcohol treatment facility / detox hospital stay	10.206	8.828	18.714	11.293	25.688	8.585
**# of residential drug or alcohol treatment facility / detox hospital stay days**	**0.692**	**3.057**	**0.516**	**3.021**	**0.341**	**0.830**
* Outpatient*						
* Hospital clinic / outpatient department*						
# of hospitalization clinic / outpatient departments visited	0.073	0.128	0.029	0.126	0.031	0.048
# of visits per hospital clinic / outpatient department	0.532	1.649	0.297	0.224	0.511	0.507
**# of hospital clinic / outpatient department visits**	**0.040**	**0.188**	**0.009**	**0.048**	**0.016**	**0.038**
* Community clinic / neighborhood health center*						
# of community clinic / neighborhood health centers visited	0.049	0.181	0.035	0.095	0.028	0.049
# of visits per community clinic / neighborhood health center	1.030	3.311	0.269	0.327	0.600	1.394
**# of community clinic / neighborhood health center visits**	**0.045**	**0.285**	**0.009**	**0.035**	**0.017**	**0.071**
* Physician*						
# of physicians visited	0.006	0.055	0.004	0.028	0.003	0.020
# of visits per physician	0.575	0.331	0.555	0.527	0.616	0.907
**# of physician visits**	**0.004**	**0.050**	**0.002**	**0.019**	**0.002**	**0.017**
* Mental healthcare providers*						
# of mental healthcare providers visited	0.056	0.131	0.023	0.103	0.049	0.079
# of visits per mental healthcare provider (psychological / emotional issues)	0.433	0.454	1.091	3.436	0.604	0.764
**# of mental healthcare provider visits (psychological / emotional issues)**	**0.029**	**0.110**	**0.017**	**0.158**	**0.029**	**0.061**
# of visits per mentalhealth care provider (medication management)	0.400	0.419	0.396	0.323	0.508	0.615
**# of mental healthcare provider visits (medication management)**	**0.021**	**0.094**	**0.011**	**0.073**	**0.020**	**0.049**
* Alcohol / drug treatment providers*						
# of alcohol / drug treatment providers visited	0.060	0.384	0.009	0.079	0.038	0.058
# of visits per alcohol / drug treatment provider	0.894	1.329	0.555	0.421	1.045	2.330
**# of alcohol / drug treatment provider visits**	**0.062**	**0.615**	**0.007**	**0.076**	**0.039**	**0.140**
# of visits per alcohol / drug treatment provider (medication management)	0.810	1.286	2.137	4.278	1.148	2.758
**# of alcohol / drug treatment provider visits (medication management)**	**0.031**	**0.159**	**0.033**	**0.576**	**0.037**	**0.140**
**Societal perspective**						
**# of days experienced alcohol problems**	**0.785**	**4.369**	**1.591**	**7.436**		
**# of days experienced drug problems**	**8.405**	**12.806**	**4.542**	**9.980**		
**Money spent on alcohol**	**$22**	**$134**	**$68**	**$618**		
**Money spent on drugs**	**$657**	**$1370**	**$200**	**$871**		

Note: A blank cell confers the measure was not asked in that study. Costs are in 2017 U.S. dollars

Missingness was low across the three studies. We categorized missing data as: 1) an absence of information or 2) invalid responses [22]. In our studies, absence of information included responses left blank and responses of “I don’t know,” “Refuse to respond,” or “N/A.” Invalid responses included out-of-range responses (e.g., 50 ED visits in 30 days), and incompatible compound responses. For questions assessing frequency and duration separately, if either question was left blank *or* if one of the two questions was answered with a positive response and the other with a zero (e.g., zero hospitalizations, 2 days each), we considered the response to that measure to be missing due to incompatability of combined responses. Invalid compound missingness was only applicable to the combined measures of utilization, whereas missingness due to absence of information or out-of-range responses was applicable to all measures. For the purpose of this study, which was limited to baseline data, we removed missing responses to individual measures from our analysis through case deletion, rather than create a complete data set through imputation.

As dictated by the healthcare sector and societal perspectives, we attempted to find MCFs designed to capture the value of the resources utilized, without accounting for other characterstics, such as profit [23]. We used the U.S. nationally-representative Medical Expenditure Panel Survey [24], to capture Medicare payments for hospital-based ED visits, hospitalization days, hospital clinic or outpatient department visits, community clinic or neighborhood health center visits, and physician visits; the Alcohol and Drug Services Study, from the Substance Abuse and Mental Health Services Administration [25], to value drug or alcohol residential treatment, detoxification hospital stays, and treatment provider visits; the Medicare physician fee schedule [26–28], to value mental healthcare provider visits; and data pooled by McCollister et al. [17], to value days experiencing alchohol or drug problems. Mean resource utilization figures were then multiplied by corresponding MCFs, converted to 2017 USD, in order to generate mean costs per 30 days at baseline for each measure across all three studies. Participant spending on substances was reported in dollar units, thus no MCFs were applied.

This study does not represent direct human subjects research and was a secondary analysis of de-identified data from STTR studies; each original study had IRB approval. This study was completed under a data sharing agreement with the STTR Data Coordination Center in which all authors agreed to respect and protect the privacy of the original participants.

## Results

Baseline healthcare resource utilization represented by varying timeframes ranging from 30 days to lifetime is presented in Table 1. Within the general medical care services domain, the studies reported utilization for the past 6 or 12 months. BRIGHT 2 had a more limited selection of outcomes as it did not ask participants about utilization of dental care, provider visits for trauma counseling, or nights spent in a homeless or emergency shelter. All studies asked about SUD treatment and whether medication was prescribed for AUD or other SUDs in the past 6 or 12 months. All studies included measures of number of times treated for AUD and other SUDs over lifetime. BRIGHT 2 respondents reported lifetime prescriptions for AUD and other SUDs, whereas PACTo and RETAIN respondents reported prescriptions received during the past 30 days. PACTo and RETAIN also asked about participant spending on substances over the past 30 days.

We calculated means and standard deviations for baseline healthcare resource utilization, per 30-day period, across the three studies (Table 2). From the healthcare sector perspective, the studies asked one common measure (number of hospital-based ED visits) in a single question. We derived the remaining nine measures by combining questions indicating frequency and duration. Other measures relevant to the societal perspective were single-question measures.

**Fig. 1:**
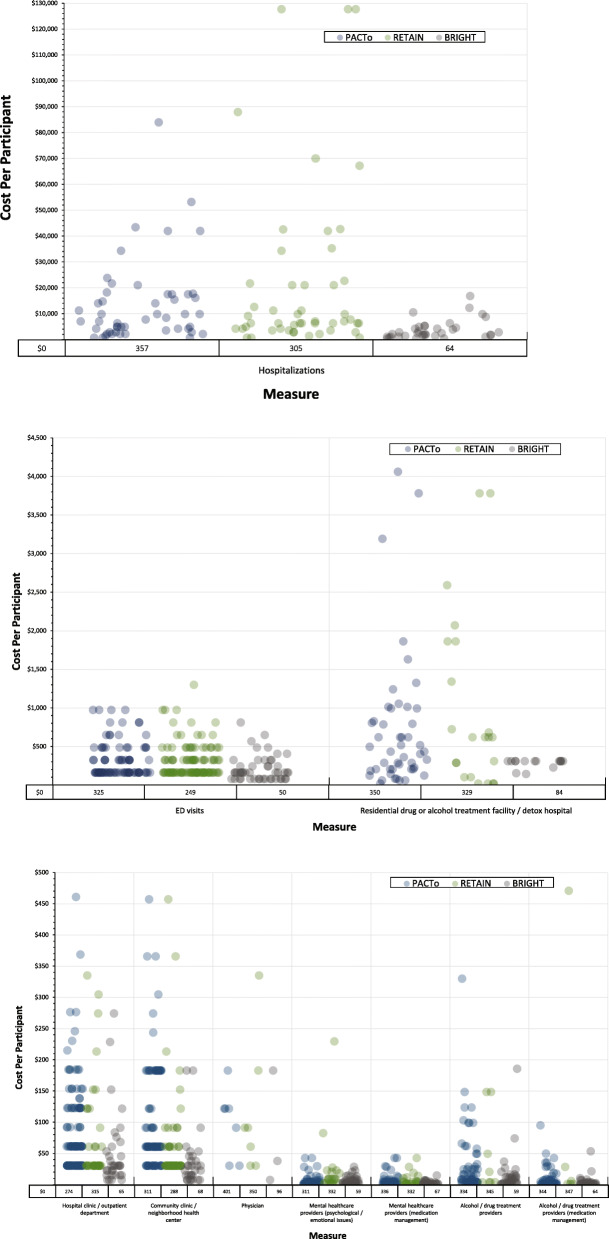
**a** Distribution of Per Participant Hospitalization Costs in Past 30 Days Across Three STTR Studies. Note: Costs are in 2017 U.S. dollars. Within each site, points representing participants are randomly placed along the x-axis for data visualization purposes. Points representing participants with costs of $0.00 are not plotted, their counts are given in the single-row table below the x-axis. **b**. Distribution of Per Participant ED Visit and Residential/Detoxification Encounter Costs in Past 30 Days Across Three STTR Studies. Note: Costs are in 2017 U.S. dollars. Within each site and measure, points representing participants are randomly placed along the x-axis for data visualization purposes. Points representing participants with costs of $0.00 are not plotted, their counts are given in the single-row table below the x-axis. **c.** Distribution of Per Participant Outpatient Costs in Past 30 Days Across Three STTR Studies. Note: Costs are in 2017 U.S. dollars. Upper 2% of data (*n* = 15) not shown. Within each site and measure, points representing participants are randomly placed along the x-axis for data visualization purposes. Points representing participants with costs of $0.00 are not plotted, their counts are given in the single-row table below the x-axis

Average baseline healthcare resource utilization costs are reported in Table 3. The average 30-day cost (per study participant) of healthcare resource utilization ranged from $1530 (BRIGHT 2) to $3347 (RETAIN). The most costly measure of healthcare resource utilization was hospitalization days, with an average 30-day cost ranging from $1332 (BRIGHT 2) to $3156 (RETAIN). The average 30-day cost of “days experiencing alcohol problems” was $15 (PACTo) and $30 (RETAIN), and the average 30-day cost of “days experiencing drug problems” was $86 (PACTo) and $160 (RETAIN). For PACTo and RETAIN, average 30-day spending on alcohol was $22 and $68, and average 30-day spending on illicit substances was $657 and $200. We show 30-day cost, per participant, of ED, inpatient, and outpatient services across the three studies (Fig. 1a, b, c).

**Table 3:** Mean Cost in Past 30 Days at Study Base line

Measures from Surveys	MCF from Literature	MCF Source	Unit Cost	Mean Cost Per Participant		
				PACTo	RETAIN	BRIGHT 2
**Healthcare sector perspective**						
Emergency department visits (hospital-based)	Emergency department visits	Medical Expenditure Panel Survey [24]	$989	$68	$98	$105
* Inpatient*						
Hospitalization days	Inpatient hospital stay (per night)	Medical Expenditure Panel Survey [24]	$4255	$1664	$3156	$1332
Residential drug or alcohol treatment / detox hospital stay days	Residential drug treatment facility (per day)	ADSS cost study (SAMHSA 1997) [25]	$126	$87	$65	$43
* Outpatient*						
Hospital clinic / outpatient department visits	Outpatient visit (community clinic or private doctor)	Medical Expenditure Panel Survey [24]	$1128	$45	$10	$18
Community clinic / neighborhood health center visits	Outpatient visit (community clinic or private doctor)	Medical Expenditure Panel Survey [24]	$1128	$51	$11	$19
Physician visits	Outpatient visit (community clinic or private doctor)	Medical Expenditure Panel Survey [24]	$1128	$4.40	$2.36	$2.31
Mental healthcare provider visits (psychological/emotional issues)	Psychologist visit	Physician Fee Schedule (Code 90834) [26–28]	$85	$2.51	$1.46	$2.45
Mental healthcare provider visits (medication management)	Psychologist visit	Physician Fee Schedule (Code 99212) [26–28]	$44	$0.92	$0.49	$0.87
Alcohol / drug treatment provider visits	Individual visit with substance use provider	ADSS cost study (SAMHSA 1997) [25]	$153	$9.52	$1.11	$5.98
Alcohol / drug treatment provider visits (medication management)	Individual visit with substance use provider	ADSS cost study (SAMHSA 1997) [25]	$44	$1.35	$1.47	$1.61
**TOTAL**				**$1934**	**$3347**	**$1530**
**Societal perspective**						
Days experiencing alcohol problems	Day experiencing psychological or psychiatric problems	McCollister, Yang [17]	$19	$15	$30	
Days experiencing drug problems	Day experiencing psychological or psychiatric problems	McCollister, Yang [17]	$19	$160	$86	
Purchase of alcohol	N/A	Self-report	N/A	$22	$68	
Purchase of drugs	N/A	Self-report	N/A	$657	$200	

Note: a blank cell confers the measure was not asked in that study. Costs are in 2017 U.S. dollars

**Fig. 2:**
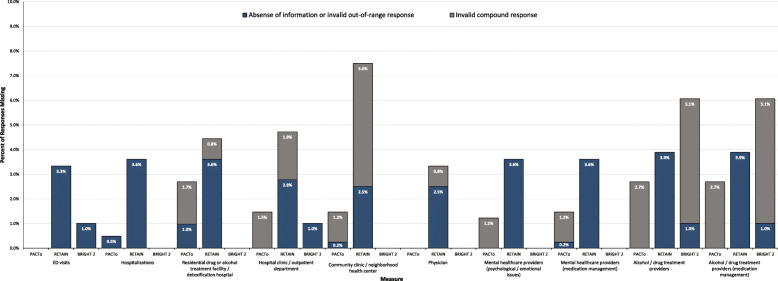
Missing Data Across Three STTR Studies. Q1 = question 1; Q2 = question 2

Given these data were taken from baseline assessments, responsiveness and completeness of data were generally high and loss to follow-up was not applicable. Rates and causes of missingness across the three studies is captured in Fig. 2, with missingness ranging from 0 to 7.5% of observations for a single measure in a given study (Additional file 1 Fig. 1 shows more detailed causes of missingness). ED visits had a rate of missingness ranging from 0% (PACTo) to 3.3% (RETAIN). Missingness for combined frequency/duration measures were as high as 5.1%, which was attributable to compound missingness in addition to missingness due to absence of information or out-of-range responses.

## Discussion

The primary objectives of this study were to review commonly collected healthcare resource utilization measures from three studies, and propose standards that would allow enhanced comparability across studies; and to report standardized healthcare resource utilization cost estimates according to different stakeholder perspectives. We identified common healthcare resource utilization measures across the three studies, all of which were reported for the past 6 or 12 months. Questions asked over a lifetime timeframe were excluded because they could not be monetized meaningfully – a discrete measure of frequency and duration is necessary in order to enumerate costs. Only one common measure (number of hospital-based ED visits) was asked as a single question. Other measures required additional effort in order to combine responses into a measure of units of service over a specific recall period. These combined frequency/duration measures of healthcare resource utilization resulted in an increased opportunity for invalid compound missingness.

Self-report is a common means of comprehensively capturing healthcare resource utilization in the absence of data from a fully integrated healthcare system or insurance claims, and has been shown to be reliable when compared to administrative and medical claims data, particularly over shorter recall periods; extrapolating these results in order to infer costs over longer timeframes is inexact and subject to recall bias [16, 29, 30]. Further consideration as to how to ameliorate the effects of recall bias while maintaining overall accuracy at baseline and follow-up is needed when selecting recall periods. Additionally, because our study was limited to baseline survey reponses, we could not evaluate differences in survey completion or reliability between baseline and treatment follow-up.

In addition to harmonization of recall timeframes, we recommend the use of single-question measures in order to minimize missingness due to incompatible compound responses. For instance, responses to the question “In the last 30 days, how many days did you spend in a residential drug or alcohol treatment facility or detox hospital?” are immediately quantifiable, as they represent a count of service utilization over a specified timeframe. While we employed case deletion for the purposes of this study, best practice is to assess the mechanism of missing data (e.g. missing at random) and employ a relevant and robust method for controlling for missing data bias (e.g., multiple imputation) [31].

These findings are meant to support ongoing data harmonization efforts in the fields of SUD, HCV, and HIV research. Helping to End Addiction Long-term (HEAL) Initiative funding opportunities supported by NIDA in response to the opioid crisis include integrated studies to develop, test, and validate evidence-based approaches to preventing and reducing opioid use disorders, overdoses, and overdose fatalities [32]. Investigators involved in these studies are expected to harmonize measures to the extent possible, in order to increase comparability of outcomes, and allow for subsequent cross-site analyses [33, 34]. Furthermore, NIDA has encouraged the incorporation of economic evaluations of study interventions from the societal and healthcare sector perspectives [33, 34]. Our results serve as an example of how harmonized healthcare resource utilization measures can be used to estimate comparable costs using standard MCFs.

Developing a common set of tools and resources for assessing utilization of healthcare resources, as well as other relevant measures such as criminal activity [35], will promote comparability of economic analysis results across studies, consistent with the recommendations of the Second Panel on Cost-Effectiveness in Health and Medicine, as well as standard methods in conducting cost-benefit analyses [4, 23, 36]. Data harmonization improves the accessibility and compatibility of data across independent studies, facilitating secondary data analyses to help answer previously unadressed or insufficiently addressed research questions regarding the most effective and cost-effective treatment paths for persons with SUDs. Robust estimates of the relative economic benefits and costs of SUD treatments and interventions helps inform the decisions of policymakers and other stakeholders tasked with balancing effectiveness and expenses as it pertains to improving the well-being of their respective populations, including persons with SUDs. Future studies should examine the implications in different health system contexts, including low and middle-income countries.

## Conclusion

Harmonizing and standardizing data measures allows for more accurate comparisons of outcomes across studies, as well as cross-study analyses. We recommend using single question measures representing units of service with specific recall periods in order to minimize missingness and generate responses that can be enumerated using standard MCFs consistent with current cost-effectiveness and cost-benefit analysis guidelines. These results can be used in cost-effectiveness and cost-benefit analyses to estimate the relative economic value of reducing healthcare resource utilization through effective interventions. Identifying the downstream economic impact of one SUD treatment or prevention strategy relative to another greatly enhances the ability of stakeholders to invest scarce resources in a manner that will allow them to reach the greatest number of those in need.

## Supplementary Information

**Additional file 1:** **Figure 1.** Causes of Missing Data Across Three STTR Studies.

## Data Availability

Individuals interested in collaborating or working with these data should contact the STTR Data Coordination Center at sttr@uw.edu.

## Abbreviations

ADSS: Alcohol and Drug Services Study
AUD: Alcohol use disorder
BRIGHT 2: Baltimore-Rhode Island Get HIV Tested
ED: Emergency department
HCV: Hepatitis C virus
NIDA: National Institute on Drug Abuse
OUD: Opioid use disorder
PACTo: Enhanced Access to HIV Care for Drug Users in San Juan, Puerto Rico
RETAIN: Providing Integrated Care for HIV-Infected Crack Cocaine Users
SAMHSA: Substance Abuse and Mental Health Services Administration
STTR: Seek, Test, Treat, and Retain
SUD: Substance use disorder
U.S.: United States
