# Supplementary material for: Harmonizing healthcare and other resource measures for evaluating economic costs in substance use disorder research
Source: Subst Abuse Treat Prev Policy. 2021 Apr 8;16:32. doi: 10.1186/s13011-021-00356-z (PMC8033702; doi:10.1186/s13011-021-00356-z)

**Additional file 1 Figure Legend**

**Additional file Figure A1.** Missing Data Sources Across Three STTR Studies.

Q1 = question 1; Q2 = question 2

**Additional file 1 Figure 1.** Causes of Missing Data Across Three STTR Studies


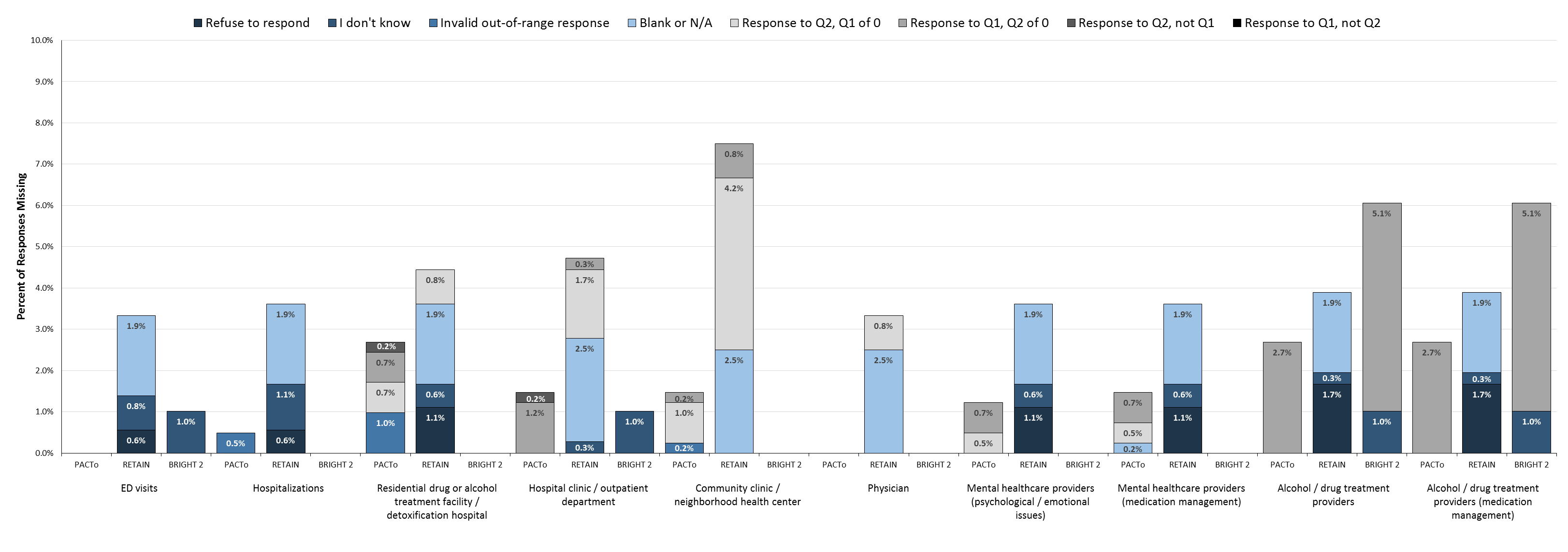

Supplement: Supplementary file 1 — Additional file 1: Figure 1. Causes of Missing Data Across Three STTR Studies. [file 13011_2021_356_MOESM1_ESM.docx]
